# The influence of recreational angling on mental health among older adults: a self-determination theory perspective

**DOI:** 10.3389/fpsyg.2025.1745450

**Published:** 2026-01-12

**Authors:** Jun Wang, Longjiang Chen, Zhengyang Mei, Xingxiao Yin, Lei Song, XianTing Zhang

**Affiliations:** 1 College of Physical Education, Yunnan Normal University, Kunming, China; 2 School of Physical Education, Southwest University, Chongqing, China; 3 Yunnan Academy of Social Sciences, Kunming, China; 4 School of Humanities and Management, Guangdong Medical University, Dongguan, China

**Keywords:** mental health, older adults, recreational angling behavior, self-determination theory, sense of well-being

## Abstract

**Objective:**

This study explored how recreational fishing influences the elderly’s psychological well-being through self-determination theory (SDT) and analyzed the mediating roles of autonomy, competence, and belonging.

**Methods:**

A total of 364 elderly anglers in Yunnan Province were investigated with the Leisure Fishing Behavior Questionnaire, Basic Psychological Needs Scale, and Index of Well-Being scale. The mediating effect of self-determination theory on recreational fishing behavior and well-being was analyzed by structural equation modeling (SEM).

**Results:**

Among 364 older recreational anglers, structural equation modeling showed that recreational angling behavior was positively associated with subjective well-being (total standardized effect = 0.17, *p* < 0.001). Autonomy, competence, and belonging partially mediated this association, accounting for approximately 21%, 14%, and 12% of the total effect, respectively.

## 1 Introduction

Against the backdrop of accelerating population aging (a demographic phenomenon characterized by an increasing proportion of elderly population and a decreasing proportion of younger population in a country or region), between 20 and 34% of older adults in 25 European countries report feelings of loneliness, compared with 25 to 29% in the United States. According to the World Health Organization’s 2021 Advocacy Brief: Social Isolation and Loneliness Among Older People, there exists a significant correlation between loneliness and health status, which will substantially impact older adults’ quality of life (an individual’s overall well-being encompassing happiness, satisfaction, and general welfare, including comprehensive assessments of health status, economic conditions, social relationships, and environmental quality) (Shekelle et al., 2024). Older adults face the combined challenges of physical decline and a diminishing social role, both of which negatively impact their psychological well-being (Liu et al., 2025). To ensure a healthy and positive aging process, individuals should adopt strategies that maintain or enhance their quality of life. Among non-pharmacological intervention strategies, recreational sports activities are widely recommended as an effective approach to improving older adults’ quality of life (Cakar and Kadioglu, 2025; Su et al., 2014).

Typically taking the form of low-to-moderate intensity leisure sports activities, recreational pursuits serve as both a vital source of social support and a cornerstone of health preservation, thereby constituting a core element of quality of life. Leisure practices such as visiting friends, engaging in recreational games, or performing daily errands not only strengthen support networks and emotional well-being but also serve as key determinants of life satisfaction (Parra-Rizo et al., 2022; Luo et al., 2024). Compared to purely physical activities, recreational fishing shows great potential for improving the mental health of older adults because it is low-pressure and immersive, in stark contrast to activities that are more physically demanding (Li et al., 2023; Liu et al., 2024). “Recreational Angling Behavior” (RAB) is defined as a non-competitive, not-for-profit outdoor recreational activity that involves relaxation of the body and mind through angling, with core characteristics including natural contact, progressive skills, and low social comparison pressure (Wilson et al., 2023). This definition aligns with established frameworks in leisure studies (Wilson et al., 2023) and incorporates key characteristics such as natural immersion, skill development, and low social comparison pressures, which are especially relevant for older adults in improving mental health and reducing stress (Li et al., 2023; Liu et al., 2024). Empirical research confirms that angling skills training significantly improves the mental health of older people, enhancing both psychological and physical well-being (Hunt and McManus, 2016). Recreational angling and participation in nature contact led to an 18.3 percent drop in cortisol in older adults, directly improving moods (Speir et al., 2020). As a low-intensity and high-participation outdoor activity, it has gradually become an important vehicle for improving the mental health of the elderly. This aligns with public policy initiatives aimed at promoting active aging and improving mental well-being in older populations, especially in the post-COVID-19 context, where social isolation and mental health concerns have become more prevalent. Governments worldwide have increasingly recognized the importance of non-pharmacological interventions like recreational activities to improve quality of life and reduce healthcare costs.

“Self-Determination Theory” (SDT), introduced by Ryan and Deci (2000b), emphasizes that the satisfaction of three basic psychological needs—autonomy, competence, and relatedness—is a key driver of intrinsic motivation and well-being. This theory provides a robust framework for understanding how recreational activities like angling can enhance the well-being of older adults by fulfilling these psychological needs. However, most of the existing studies explain the benefits of angling based on the stress-relieving model and lack an in-depth analysis of the mechanism of psychological need satisfaction (Lam, 2024). Although Self-Determination Theory (SDT) is widely used in sport psychology (Sheeran et al., 2020), most studies have focused on group-based leisure activities (e.g., square dancing and tai chi) and have rarely examined the mechanism of angling, which combines individual immersion and social attributes (Zhang et al., 2010); therefore, theory-oriented mechanism analyses remain scarce. Therefore, this paper explores the mediating role of self-determination theory in leisure fishing behavior and elderly well-being, and systematically analyzes the mechanism of leisure fishing behavior on elderly mental health, focusing on two limitations. First, break through the limitations of the traditional single explanatory path model and turn to the research on the process mechanism of psychological need satisfaction. Secondly, it is to fill the gap of self-determination theory in the analysis of psychological mechanism of the elderly in fishing leisure activities. Clarify the mediating effects of autonomy, competence and belonging, and provide theoretical basis for the construction of aged-friendly non-drug intervention programs.

Self-Determination Theory (SDT) emphasized that the core of human behavioral motivation lies in the satisfaction of three basic psychological needs. First, autonomy: the individual’s perception that behavioral choices are self-directed rather than dictated by external pressures. Critically, competence: the individual’s experience of increased competence and goal attainment in activities. Finally, relatedness: the individual’s ability to build meaningful social connections and emotional resonance with others. SDT suggests that when these three types of needs are met, individuals will develop intrinsic motivation, which in turn promotes mental health and well-being (Ryan and Deci, 2000a). The theory has been widely used in the field of sport psychology, confirming its ability to explain the mechanisms by which leisure activities promote mental health (Ng et al., 2012). This complements other recent frameworks in “gerontological psychology,” including “compensatory selective optimization” (Li et al., 2022), which emphasizes how older adults can maximize their strengths and minimize losses; and “activity theory” (Adelmann, 1994; Lam and García-Román, 2020), which posits that staying active is key to maintaining well-being. In terms of autonomy, angling activities allow older people to decide when, where, and how to participate (e.g., wild/pond angling, alone/partnered), and this sense of control over the environment is at the heart of the need for autonomy (Zhao et al., 2016). Studies have shown that older people who select leisure activities by themselves directly improve their well-being by lowering cortisol levels through self-selected exposure to the natural environment (Bratman et al., 2015b). It also reduces the risk of depression; in terms of competence, recreational angling requires skills such as fish habit identification and tackle operation, and older people develop a sense of self-efficacy through progressive learning. Liu et al. (2024) noted that natural immersion and skill development are beneficial for enhancing participants’ subjective well-being in outdoor activities. Empirical evidence suggests that recreational outdoor exercise can enhance the mental and cognitive abilities of older adults (Emery and Blumenthal, 1991). In addition, skill acquisition enhances the self-efficacy and life satisfaction of older persons (Lyubomirsky et al., 2005). Concerning the sense of belonging, angling activities are often accompanied by social interactions such as exchanges among angling buddies and experience sharing to enhance the sense of belonging and effectively alleviate social isolation among older people. The study found that the loneliness of older adults who participated in collective social leisure activities could be effectively reduced (Griffiths et al., 2017). It has been shown that participation in recreational angling is associated with lower levels of loneliness and higher levels of well-being (Trott et al., 2023). Specifically, recreational angling can positively influence well-being and older adults’ sense of autonomy and competence, and sense of belonging through angling can contribute to their well-being.

This study aims to examine how Self-Determination Theory (SDT) mediates the relationship between Recreational Angling Behavior and Subjective Well-being in older adults. Specifically, it will assess the roles of autonomy, competence, and relatedness in enhancing well-being through recreational angling. Based on this, this study proposes the following hypotheses: **H1**: Recreational Angling Behavior positively predicts older people’s Subjective Well-being; **H2**: Self-determination theory mediates Recreational Angling Behavior and older people’s Subjective Well-being.

## 2 Methods

### 2.1 Research design

This study adopted an observational, cross-sectional survey design. All variables were measured once using self-report questionnaires administered to older adults engaged in recreational angling in natural fishing environments. Based on self-determination theory, structural equation modeling (SEM) was used to test the hypothesized mediation model linking recreational angling behavior, basic psychological needs (autonomy, competence, and relatedness), and subjective well-being.

### 2.2 Participants

Prior to data collection, the study protocol was reviewed and approved by the Ethics Committee of Yunnan Normal University (Approval No. ynunethic2025-065). All procedures complied with the Declaration of Helsinki and relevant institutional guidelines. Given the exploratory nature of the study and the need to feasibly reach older adults in natural fishing environments, purposive and convenience sampling methods were employed. These approaches enabled us to target older adults engaged in recreational angling across diverse real-world settings, thereby enhancing the ecological validity of the findings.

We collected multi-site data on recreational angling activities in the central Yunnan region. The study area covered three typical types of angling environments: municipal planned angling areas (public welfare urban waters), rural reservoirs, and legally compliant commercial angling grounds, geographically spanning five cities and 11 counties.

The inclusion criteria required participants to be 60 years of age or older and to have at least 1 year of recreational angling experience. We excluded individuals with diagnosed cognitive impairment or severe physical limitations that could interfere with fishing participation or questionnaire completion, as well as those who did not meet the specified age or angling experience requirements. Only participants who satisfied all inclusion criteria and none of the exclusion criteria were invited to complete the survey.

At each study site, trained investigators approached older adults who were actively engaged in angling during the survey period. The investigators first provided a brief, standardized explanation of the study aims, procedures, voluntary nature of participation, and expected duration of the survey. Potential participants received an information sheet describing the study, their rights (including the right to refuse or withdraw at any time without penalty), and key aspects of data privacy and confidentiality. Those who agreed to participate signed a written informed consent form before completing the questionnaire. Regarding data protection, no names, phone numbers, or other direct personal identifiers were recorded on the questionnaires. For participants with limited literacy, the investigators read the information and consent form aloud in clear language and confirmed understanding before obtaining written consent.

To maximize participation while minimizing disruption to angling activities, data collection followed a two-phase time-locking strategy focused on intensive daytime angling hours (06:00–09:00 in the morning and 16:00–20:00 at dusk), which is consistent with the daily routines of the elderly population and helps improve response rates. The fieldwork period spanned from August 2024 (rainy season) to April 2025 (spring hydrological cycle).

### 2.3 Measurements

The Recreational Angling Behavior Questionnaire was adapted from the Recreational Engagement Scale (RES) developed by Şimşek and Çevik (2020), The scale consists of 9 items measuring eight dimensions, namely, relaxing, developmental, socializing, activity with an attractive environment, productive, esthetic, entertaining, and exciting activity, with 2 items per dimension. For example: “I find angling relaxing.” A 7-point Likert scale was used, ranging from 1 for ‘do not agree’ to 7 for ‘agree very much’, and a reverse questionnaire was used intermittently to increase the reliability of the study, which was found to have a factor loading of less than 0.4 and was discarded during the reliability analysis, resulting in a total of 9 items. The alpha coefficient of the total questionnaire was 0.870, and the coefficient of the KMO test was 0.932. The results of the validated factor analysis were: *χ*^2^/df = 0.665, RMSEA = 0.000, SRMR = 0.021, GFI = 0.999, NFI = 0.984, CFI = 1.000, IFI = 1.008.

The well-being questionnaire was based on the Subjective Well-Being Scale (SWB) developed by Diener et al. (1985). The scale consists of 25 items measuring two dimensions: cognitive and affective dimensions, and includes 5 questions on life satisfaction and 20 questions on positive and negative affect. For example: “I feel satisfied with my life.” A 7-point Likert scale was used, ranging from 1 for “do not agree” to 7 for “strongly agree”, and a reverse questionnaire was used intermittently to increase the reliability of the study. The alpha coefficient of the total questionnaire was 0.905, and the coefficient of the KMO test was 0.945. The results of the validated factor analyses were: *χ*^2^/df = 1.130, RMSEA = 0.019, SRMR = 0.038, GFI = 0.988, NFI = 0.871, CFI = 0.983, and IFI = 0.983.

This study employed the questionnaire developed by Chen et al. (2015) based on Self-Determination Theory. The questionnaire consists of 12 items derived from the Basic Psychological Needs Scale (BPNS), which encompasses three dimensions: autonomy, competence, and relatedness. Each dimension has 4 question items. For example: “I feel free to choose when and where I engage in angling.” A 7-point Likert scale was used, ranging from 1 for ‘do not agree’ to 7 for ‘agree very much’, and a reverse questionnaire was used intermittently to increase the reliability of the study. The alpha coefficient of the total questionnaire was 0.649, and the coefficient of the KMO test was 0.717. The results of the validated factor analyses were: *χ*^2^/df = 1.129, RMSEA = 0.019, SRMR = 0.025, GFI = 0.995, NFI = 0.946, CFI = 0.993, and IFI = 0.993. In this study, the Cronbach’s alpha for the SDT scale was 0.649, which is slightly below the conventional 0.70 benchmark but may be considered acceptable for a brief, multidimensional scale with four items per subscale. Together with the satisfactory CFA indices, these results indicate that the scale has acceptable reliability and construct validity for use in this exploratory study.

To improve the reliability of the study and minimize response bias, reverse coding items were included in the SDT (3 items), recreational fishing behavior (2 items), and subjective well-being scales (4 items). Despite the inclusion of inverse-coded items, the factor structure is preserved. Inclusion of these items helps ensure that participants carefully consider their answers and reduces the likelihood of response bias.

### 2.4 Test procedures and data processing

At the initiation stage of questionnaire completion, the informed consent document should be signed by the subjects, and the research instrument should be filled out anonymously. Before the formal study, the principal examiner should explain the principle of strict confidentiality of the data to the interviewees, affirming that the content of the collection is limited to the use of academic research. Requesting the subjects to complete all the entries independently and conscientiously following the instruction specifications. If you have difficulty filling in, volunteer to assist in filling in. The data collection process lasted about 15 min or more, and the retrieval process was completed uniformly at the site. First, confirmatory factor analyses (CFAs) were conducted within a structural equation modeling (SEM) framework to validate the measurement models of recreational angling behavior, autonomy, competence, belongingness, and well-being. After acceptable model fit was confirmed, the hypothesized mediation model was tested using the PROCESS macro (Model 4) with 5,000 bootstrap samples and 95% confidence intervals in SPSS 26.0. The PROCESS results served as the primary basis for interpreting the mediation effects.

## 3 Results

### 3.1 Common methodological biases

Since all variables in this study were derived from participants’ self-reports, common method bias may be a concern. To mitigate this risk, we adopted several procedural remedies during data collection. First, items from different constructs were presented on separate pages to reduce response sets and mechanical answering. Second, a mandatory 15-s dwell time was implemented between pages to alleviate response inertia and encourage more careful reading and responding. In addition, questionnaires were completed anonymously, and participants were informed that there were no right or wrong answers, which helped reduce evaluation apprehension and social desirability bias. To further assess the extent of common method bias, we conducted an exploratory factor analysis using Harman’s single-factor test. Ten latent factors with eigenvalues greater than 1 were extracted, and the first unrotated factor accounted for only 19.17% of the total variance, which is below the commonly used 40% threshold (Hair et al., 2014). These results suggest that common method bias is unlikely to have seriously distorted the observed relationships among the study variables.

### 3.2 Descriptive statistics and correlation analysis

A dual response-screening framework was used: 429 questionnaires were initially collected, and 364 valid cases were retained after stringent quality control procedures (e.g., excluding responses with unrealistically short completion times, angling experience < 1 year, or age <60). Of the 364 valid participants, 272 were male (74.7%) and 92 were female (25.3%). This gender imbalance may reflect the general demographics of recreational anglers in the region, where angling is more popular among males. However, this distribution may also present a potential sampling limitation, and we acknowledge the possibility of gender-related bias in the study. In terms of age, 237 participants (65.1%) were 60–69 years old, and 127 (34.9%) were 70 years or older. With respect to angling experience, 158 participants (43.4%) had 0.5–2 years of experience, 145 (39.8%) had 3–5 years, and 61 (16.8%) had more than 5 years of angling experience.

Spearman’s rank-order correlation coefficients among subjective well-being, recreational angling behavior, autonomy, competence, and relatedness were calculated, as shown in Table 1. Subjective well-being was significantly and positively correlated with recreational angling behavior, autonomy, competence, and relatedness at the 0.01 level. The significant correlations among the study variables provide a prerequisite for the subsequent mediation analyses.

**Table 1:** Descriptive statistics and correlation analysis (n = 364).

Variables	1	2	3	4	5
1. SWB	1				
2. RAB	0.22***	1			
3. Autonomy	0.26***	0.23***	1		
4. Competence	0.26***	0.16**	0.04	1	
5. Relatedness	0.31***	0.11*	0.12*	0.16**	1
M	2.97	3.75	3.61	3.12	2.71
SD	0.43	0.57	0.55	0.55	0.64

*denotes *p* < 0.05, **denotes *p* < 0.01, ***denotes *p* < 0.001. SWB, Subjective Well-Being; RAB, Recreational Angling Behavior; M, Mean; SD, Standard Deviation.

### 3.3 Mediation analysis

**Figure 1:**
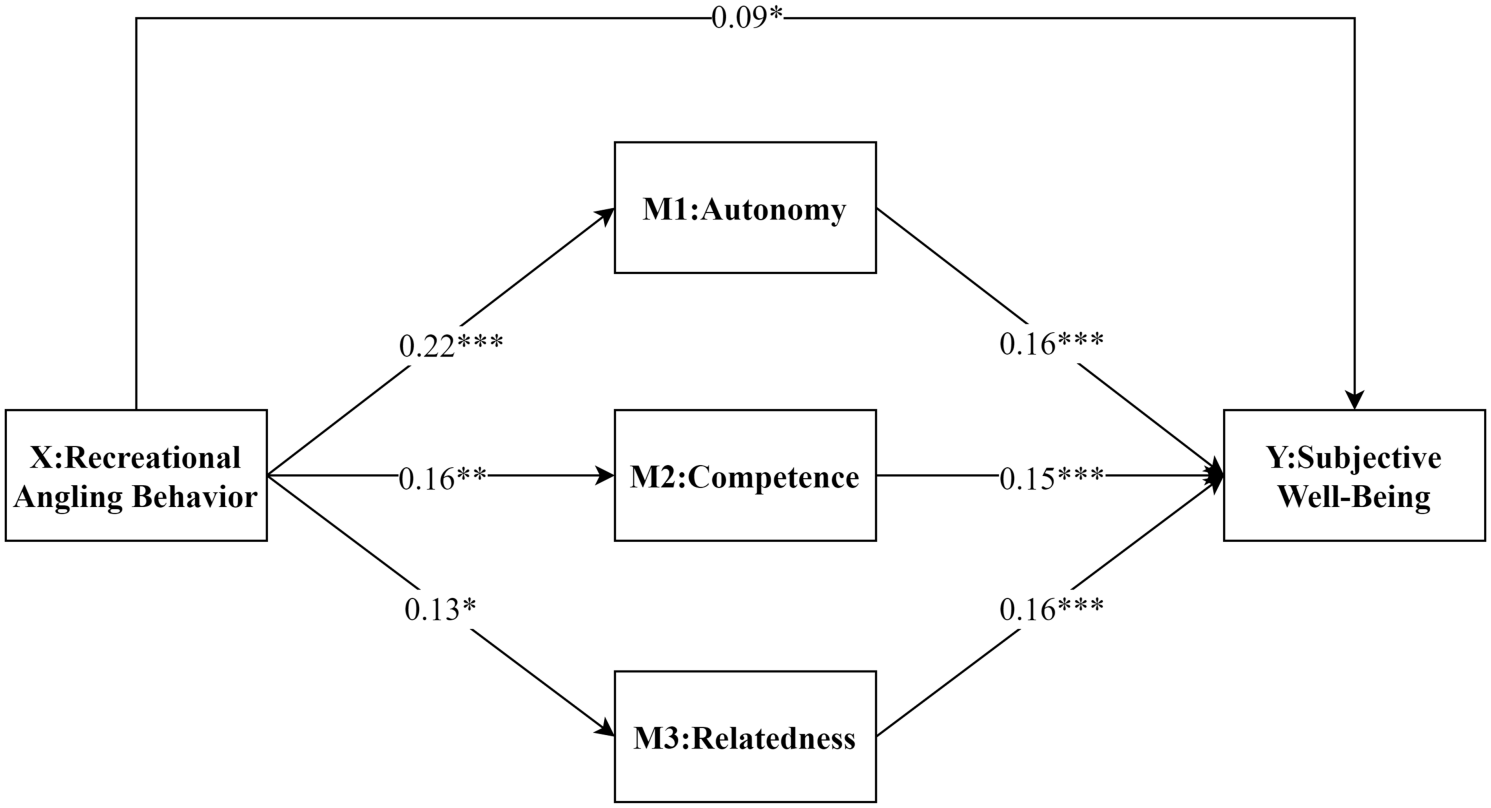
A model of the mediating role of self-determination theory in the relationship between recreational angling behavior and older adults’ well-being.

To examine whether self-determination theory variables played a mediating role in the relationship between recreational angling behavior and subjective well-being in older adults, we conducted a mediation analysis using the PROCESS macro (Model 4) for SPSS, applying a bias-corrected bootstrap procedure with 5,000 resamples (Efron, 1987; Hayes, 2013). Figure 1 and Table 2 show that the total effect of recreational angling behavior on subjective well-being was 0.17, with a 95% confidence interval of [0.10, 0.25] that did not include zero, indicating a significant total effect. The direct effect was 0.09, 95% CI [0.02, 0.16], also significant, accounting for 53% of the total effect. The specific indirect effects through autonomy, competence and relatedness were 0.04, 0.02 and 0.02, respectively, and all of their 95% bootstrap confidence intervals excluded zero, indicating significant mediation.

For descriptive purposes, we calculated the proportion of the total effect explained by each pathway by dividing each unstandardized effect by the total effect (see the “Effect size proportion” column in Table 2). In this metric, the direct path accounts for 53% of the total effect, whereas the indirect paths via autonomy, competence and relatedness account for approximately 21, 14 and 12% of the total effect, respectively. In practical terms, this means that about half of the association between recreational angling behavior and subjective well-being is direct, and the remaining part is transmitted through basic psychological needs, with autonomy representing the strongest indirect pathway.

**Table 2:** Bootstrap mediation affects test results.

Path	Effects	SE	*t*	95% CI		Effect size proportion
				BootLLCI	BootULCI	
Total effect	0.17	0.04	4.38	0.10	0.25	1
Direct effect	0.09	0.04	2.43	0.02	0.16	0.53
RAB → Autonomy → SWB	0.04			0.01	0.06	0.21
RAB → Competence → SWB	0.02			0.01	0.04	0.14
RAB → Relatedness → SWB	0.02			0.00	0.04	0.12

SWB, Subjective Well-Being; RAB, Recreational Angling Behavior; SE, Standard Error; BootLLCI, Lower Limit Confidence Interval; BootULCI, Upper Limit Confidence Interval.

## 4 Discussion

### 4.1 Direct effects of recreational angling behavior

The present study indicated that recreational angling behavior had a significant positive total effect on the psychological well-being of older adults (*β* = 0.224, 95% CI [0.13, 0.33], *p* < 0.001). This result supports our first hypothesis and shows that non-competitive, low-intensity recreational activities are good for well-being in later life. In line with past research, contact with natural blue–green settings has been associated with lowered stress biomarkers and improved moods for older adults (Bratman et al., 2015a, 2015b; Jarosz, 2023; Liu et al., 2024). According to self-determination theory, recreational angling is especially advantageous because it gives older adults the option to go at their own speed, choose voluntarily when and where to take part, and acquire skills without much social comparison pressure. Together they make a context which is appropriate for both physical and psychological need of the elderly people so as to enhance the mental health.

### 4.2 A study of the mediating role of autonomy, competence, and relatedness

This study verifies that autonomy, competence, and relatedness have considerable roles as a mediator in the relationship among leisure angling behavior and happiness for the elderly group, with autonomy as the main mediator. In particular, the autonomy accounts for 21% of the total effect (indirect effect = 0.04) compared to 14% and 12% of the total effect (indirect effects = 0.02 and 0.02) competence and relatedness, respectively. These results suggest a partially mediated relationship with recreational angling impacting well-being directly and through the satisfaction of basic psychological needs. These findings are in line with the main proposition of self-determination theory, that satisfaction of the basic psychological needs for autonomy, competence, and relatedness is an essential mechanism through which everyday activities enhance well-being.

Autonomy became the strongest mediator. That the bigger effects size means that the where,when or how you go on an angling adventure matters to explain the good vibes of fishing. Recreational angling in SDT terms provides older adults with high levels of volitional choice and personal control in their engagement in angling, thereby enhancing the sense of self-determination and psychological ownership of the activity. The previous studies have found that the self-chosen leisure activities and self-directed exposure to the natural environment can significantly reduce the psychological stress and depressive symptoms of the older people (Bratman et al., 2015a, 2015b; Luo et al., 2024). Our findings build upon this kind of proof by bringing to the fore that inside a particular situation of recreational angling, the sense of freedom is not only valuable of itself, but also serves as a meaningful route to turning the actual participation into well-being in angling.

Competence had a small, yet significant, mediating effect. Recreational angling needs various skills, like recognizing patterns in fish behavior, choosing suitable equipment, and adapting techniques according to environmental conditions. Through progressive learning and doing well, older adults can feel like they have mastered something or achieved something. This feeling of achievement can lead to increased self-efficacy and positive emotions (Bandura, 1977; Grass et al., 2023). The findings match current research that partaking in activities that use cognitive and technical skills will maintain executive function and mental health in the later years of life (Annas, 2004; Emery and Blumenthal, 1991). But, in this study the mediating effect of competence is less than the mediating effect of autonomy. According to the self-determination theory, this pattern reflects the fact that recreational fishing of older adults is mainly an environment with low external pressure and high recovery value, rather than an environment where performance and results are valued. In these places, it feels better to be free to decide when, where, and how to get involved than to be getting better and better at things all the time (Bölenius et al., 2023; Chiviacowsky and Lessa, 2017). Having achieved a basic sense of effectiveness, further gains in competence contribute relatively little more benefit than that provided by the powerful sense of autonomy. This may be one reason why competence contributes to less of the overall effect.

Relatedness explained the least of the total effect, yet it still played a considerable mediating role. The leisurely angling may involve informal communications and exchanges of experiences with other anglers and mutual assistance to lessen the feeling of solitude and improve social ties amongst older adults (Griffiths et al., 2017; Wilson et al., 2023). The relatively small effect size of this mediation might reflect individual variation in how much one needs to interact with others and that you can enjoy angling either alone or with others. Some older adults find that quiet, solitary engagement with nature is more important for their well-being than frequent social interactions. Under the SDT framework, this means that while relatedness needs can be met by angling, they can probably be experienced in a more flexible, non-essential manner compared to autonomy, and that could explain its lesser mediating role.

To conclude, the mediating pattern from this research shows that non-competitive recreational angling can have a great impact on the wellbeing of older people and that the main mechanism behind this phenomenon is their autonomy, followed by competence and finally, by relatedness. These results underscore the necessity of developing leisure environments and programs that emphasize older adults’ freedom of choice and opportunities for skill development over simply increasing the duration of activity. In general, the current results support self-determination theory by showing that the psychological benefits of recreational angling are largely due to satisfaction of basic psychological needs, particularly autonomy.

### 4.3 Research implications

This study delves into how recreational fishing behavior influences the well-being of older adults through Self-Determination Theory (SDT), addressing a theoretical gap in existing literature regarding non-competitive, immersive individual leisure activities. Specifically, it further elucidates the mediating roles of autonomy, competence, and relatedness in this process, enriching the application of SDT in the field of mental health among the elderly. Moreover, the findings contribute to a deeper understanding of SDT, particularly in how fulfilling basic psychological needs enhances older adults’ well-being, offering novel theoretical perspectives and empirical support.

At the practical level, this study provides a theoretical basis for formulating non-drug intervention programs for the elderly. Research shows that enhancing the autonomy and competence of the elderly can effectively improve their happiness. Therefore, policymakers and social organizations should pay more attention to how to design flexible and personalized leisure activities to meet the psychological needs of the elderly. In practice, skills training, social interaction platforms, and customized activity schedules can be set up to help older people enhance their self-efficacy and sense of belonging, thus improving their mental health. Local governments could collaborate with social service organizations to offer subsidized angling programs that target older adults in rural or underserved areas. In addition, this study highlights the need for targeted interventions, suggesting that policymakers integrate self-determination theory into activity planning for older adults, rather than just extending activity time, to achieve more efficient happiness.

### 4.4 Limitations and prospects

The following methodological limitations exist in this study. Cross-sectional Design: A key limitation of the present study is its cross-sectional design, which prevents establishing causal pathways between angling behavior and well-being. Future longitudinal or experimental studies are needed to assess temporal dynamics and causal directions. Additionally, Ecological Variables: environmental variables such as water quality, noise, and weather conditions should be incorporated into future models to better capture ecological moderators. In addition, Sample Limitations: The sample was predominantly male (74.7%), and the male–female ratio bias may not be fully representative of the broader population of older people engaged in recreational fishing. Finally, geographical and cultural background constraints: sample selection is limited to fishing enthusiasts in Yunnan Province, does not include the activity patterns unique to elderly anglers in coastal areas (such as Xiamen and Qingdao), and does not study the cultural background of the sample, so future research needs regional diversity to explore the role of cultural factors in psychological support pathways.

## 5 Conclusion

This study shows that recreational fishing significantly improves mental health in older adults by satisfying key psychological needs identified in the Self-Determination Theory (SDT). Specifically, autonomy is the most prominent mediator in this relationship, while competence and belonging contribute relatively little. The results suggest that interventions centered on enhancing the autonomy of activity choice development are most effective in improving well-being. The study emphasized that simply extending the duration of fishing activity was far less effective than targeted interventions targeting the psychological needs of older adults. This suggests that personalized, flexible entertainment solutions are more important than one-size-fits-all approaches. Future ageing population projects should incorporate recreational fishing into cost-effective interventions to improve mental health and social participation. By incorporating elements that support autonomy, competence and belonging, these programs can effectively improve the quality of life of older adults.

## Data Availability

The original contributions presented in the study are included in the article/supplementary material, further inquiries can be directed to the corresponding authors.
